# Characterization of a FourU RNA Thermometer in the 5′ Untranslated Region of Autolysin Gene *blyA* in the *Bacillus subtilis* 168 Prophage SPβ

**DOI:** 10.1021/acs.biochem.3c00368

**Published:** 2023-09-12

**Authors:** Alina Y. Tong, Emma E. Caudill, Alexis R. Jones, Luiz F. M. Passalacqua, Michael M. Abdelsayed

**Affiliations:** † Department of Biology, California Lutheran University, Thousand Oaks, California 91360, United States; ‡ Laboratory of Nucleic Acids, National Heart, Lung, and Blood Institute, National Institutes of Health, Bethesda, Maryland 20892, United States

## Abstract

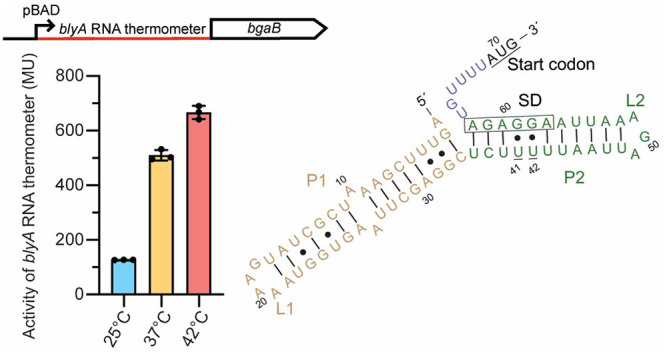

RNA thermometers are noncoding RNA structures located in the 5′ untranslated regions (UTRs) of genes that regulate gene expression through temperature-dependent conformational changes. The fourU class of RNA thermometers contains a specific motif in which four consecutive uracil nucleotides are predicted to base pair with the Shine-Dalgarno (SD) sequence in a stem. We employed a bioinformatic search to discover a fourU RNA thermometer in the 5′-UTR of the *blyA* gene of the *Bacillus subtilis* phage SPβc2, a bacteriophage that infects *B. subtilis* 168. *blyA* encodes an autolysin enzyme, *N*-acetylmuramoyl-l-alanine amidase, which is involved in the lytic life cycle of the SPβ prophage. We have biochemically validated the predicted RNA thermometer in the 5′-UTR of the *blyA* gene. Our study suggests that RNA thermometers may play an underappreciated yet critical role in the lytic life cycle of bacteriophages.

RNA thermometers are mostly *cis*-acting RNA structural elements found in the 5′ untranslated region (5′-UTR) of a gene.^1,2^ Typically, RNA thermometers regulate gene expression within a physiologically relevant temperature range of 25–42 °C. In most characterized examples, they result in an increased level of gene expression in response to higher temperatures. This temperature-dependent upregulation occurs as the RNA structure becomes denatured and reveals a previously sequestered gene expression platform.^1,2^ FourU thermometers make up a well-characterized class of RNA thermometers containing a specific motif in which the ribosome-binding site, the SD sequence, is predicted to base pair in a stem across from four consecutive uracil nucleotides.^3^ With an increase in temperature, base pairing within the fourU motif is disrupted, causing destabilization of the stem and increased accessibility to the SD sequence, enabling translation of a downstream gene.^3^ Several fourU RNA thermometers have been found in the 5′-UTR of genes involved in the heat shock response or bacterial virulence. These include the *ompA* gene in *Yersinia pseudotuberculosis*(4) and *Shigella dysenteriae*,^5^ the *agsA* gene in *Salmonella enterica*,^3^ the *toxT* gene in *Vibrio cholerae*,^6^ the *shuA* gene in *S. dysenteriae*,^7^ and the recently discovered glycerol permease fourU RNA thermometer (*glpT*) in *Bacillus subtilis.*(8)*B. subtilis* also has a second glycerol permease RNA thermometer (*glpF*) that is a non-fourU RNA thermometer.^8^

The homology among several previously characterized fourU RNA thermometer motifs was exploited to search for novel fourU RNA thermometers. The RNA motif search tool, RNArobo,^9^ was used to identify potential fourU RNA thermometers based on the predicted secondary structure of the fourU *agsA* RNA thermometer^3^ (Methods in the Supporting Information). The results of our *in silico* approach revealed a potential RNA thermometer in the 5′-UTR of the *blyA* gene (also known as *yomC*), which encodes an autolysin enzyme (*N*-acetylmuramoyl-l-alanine amidase) that facilitates the hydrolysis of cell wall glycopeptides^10^ (Figure 1A,B). This gene is found in the genomes of the *B. subtilis* phage SPβc2 (SPβ) and its host, the Gram-positive bacterium *B. subtilis 168* (*B. subtilis*). Previously, only one RNA thermometer has been described in a phage genome.^22^

**Figure 1:**
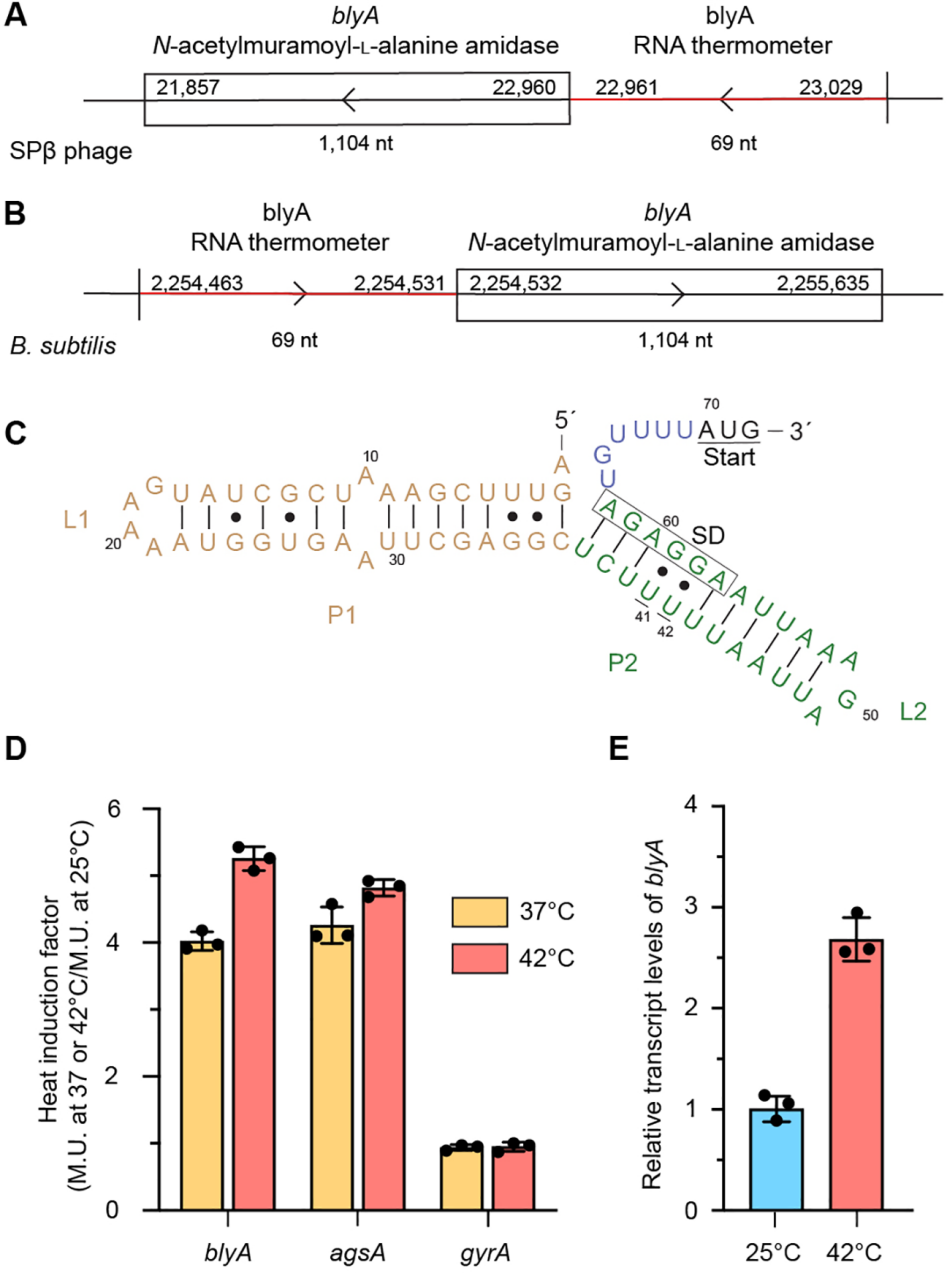
Discovery of an RNA thermometer upstream of *blyA*. Genome loci of (A) *B. subtilis* SPβ bacteriophage and (B) *B. subtilis* 168 showing the RNA thermometer upstream of the gene *blyA* that encodes an *N*-acetylmuramoyl-l-alanine amidase. (C) Secondary structure prediction of the 5′-UTR of *blyA*. The fourU U41 and U42 residues and start codon are underlined, and the Shine-Dalgarno (SD) sequence is boxed. (D) Heat induction factor of *bgaB* fusions with the *blyA* 5′-UTR. Expression at 25, 37, and 42 °C was compared to a positive control, the *agsA* fourU RNA thermometer, and a negative control, DNA gyrase (*gyrA*) (mean ± standard deviation; *n* = 3 biological replicates). (E) Relative transcript levels of the 5′-UTR of *blyA* at 25 and 42 °C measured by qRT-PCR. The transcript levels of the 5′-UTR of *blyA* were normalized to reference gene *gyrA* (mean ± standard deviation; *n* = 3 biological replicates, each with 3 technical replicates).

The prophage SPβ contains the open reading frame for *blyA*, which plays a crucial role in the lytic activity of the phage in the host cell.^10^ SPβ integrates into the genome of its bacterial host and has been found to be ubiquitous in *B. subtilis* 168.^11,12^ SPβ is a lysogenic phage with a temperature-dependent lytic life cycle in *B. subtilis* 168.^10,13,14^ Previous studies have demonstrated that the level of expression of *blyA* in *B. subtilis* 168 increases at high temperatures, and its heat-induced expression is necessary for the lysis of the host cell.^10^ Although it is known that heat stress induces the expression of *blyA* and the proliferation of the SPβ prophage in *B. subtilis* 168,^10^ the underlying molecular mechanism that drives this process has yet to be elucidated.

RNA thermometer function is dependent upon a conformational change in the RNA secondary structure. RNAfold^15^ was employed to predict the full-length structure of the RNA thermometer sequence in the 5′-UTR of *blyA* (Figure 1C). The predicted secondary structure of the 5′-UTR of *blyA* is closely related to the predicted structure of the *agsA* RNA thermometer^3^ and contains two hairpins with the fourU motif in the P2 stem. To determine the thermoregulatory function, the 5′-UTR of *blyA* (5UTR_blyA) was cloned into a reporter plasmid containing an arabinose-inducible pBAD promoter upstream of a heat-stable β-galactosidase (*bgaB*) from *Bacillus stearothermophilus.*(16) The 5UTR_blyA*–bgaB* fusion was constructed by replacing the native 5′-UTR of *bgaB* with the 5′-UTR of *blyA* (Figure S1).

Temperature-dependent expression was determined by heat induction of *Escherichia coli* cells expressing the 5UTR_blyA*–bgaB* fusion. Cells containing 5UTR_blyA*–bgaB* fusions were incubated for 30 min at 25, 37, or 42 °C. Subsequently, β-galactosidase activity was measured for each temperature (Methods in the Supporting Information). Heat shock of cells expressing 5UTR_blyA*-bgaB* fusions resulted in heat induction factors [activity in Miller units (M.U.) at 37 °C/25 or 42 °C/25 °C] of ∼4.0- and ∼5.2-fold at 37 and 42 °C, respectively (Figure 1D). *bgaB* fusions containing the established *agsA* RNA thermometer were used as positive controls and demonstrated a similar heat induction profile. As a negative control, the 5′-UTR of a DNA gyrase gene (*gyrA*), which is not expected to be thermally regulated, was tested and exhibited minimal heat induction of 0.94- and 0.95-fold at 37 and 42 °C, respectively (Figure 1D). These findings demonstrate that the 5′-UTR of *blyA* modulates reporter gene activity in a temperature-dependent manner, with a notable increase in the level of gene expression at increased temperatures.

To investigate differences between transcriptional and translational control in the system, transcript levels of 5UTR_blyA–*bgaB* fusions were measured by quantitative real-time PCR (qRT-PCR). Cells were harvested under the same conditions of β-galactosidase assays at 25 and 42 °C. Heat induction resulted in an approximately 2.6-fold increase in transcript abundance at 42 °C, indicating some regulation at the transcriptional level (Figure 1E). Similar increases in transcript levels have been reported for other RNA thermometers, suggesting an additional layer of regulation.^17,18^ It is currently unknown how transcription of the 5′-UTR of *blyA* is regulated.

To further validate that the *blyA* RNA thermometer sequence is directly responsible for the thermal regulation of gene expression, mutations were made to strengthen and stabilize the base pairing of the fourU stem motif. As previously demonstrated with the *agsA* RNA thermometer,^3^ stabilization of the fourU motif results in a decrease in the level of heat induction. The predicted secondary structure of the *blyA* RNA thermometer was used to design stabilizing mutations to the fourU motif to strengthen the base pairing of P2 (Figure 2A), and β-galactosidase assays of mutants were performed at 25 and 42 °C to determine activity. U41C and U42C mutations in the fourU region across from the SD sequence change G·U wobble base pairs to stronger canonical G-C base pairs. Strikingly, double mutant UU4142CC completely abolished heat induction activity. The U41C stabilizing mutation by itself did not show any decrease in the level of heat induction, while the mutants U42C and CUU394041AAA notably reduced the level of heat induction to ∼1.5-fold (Figure 2B). In addition, thermal analysis comparison of the wild-type 5′-UTR *blyA* sequence and the double mutant UU4142CC showed that the double mutant’s overall melting temperature (75.9 ± 0.4 °C) is considerably higher than that of the wild type (69.4 ± 0.4 °C) (Figure 2C). Altogether, our results indicate that the thermoregulation of the *blyA* RNA thermometer is directly dependent on the thermal stability of the fourU motif of P2.

**Figure 2:**
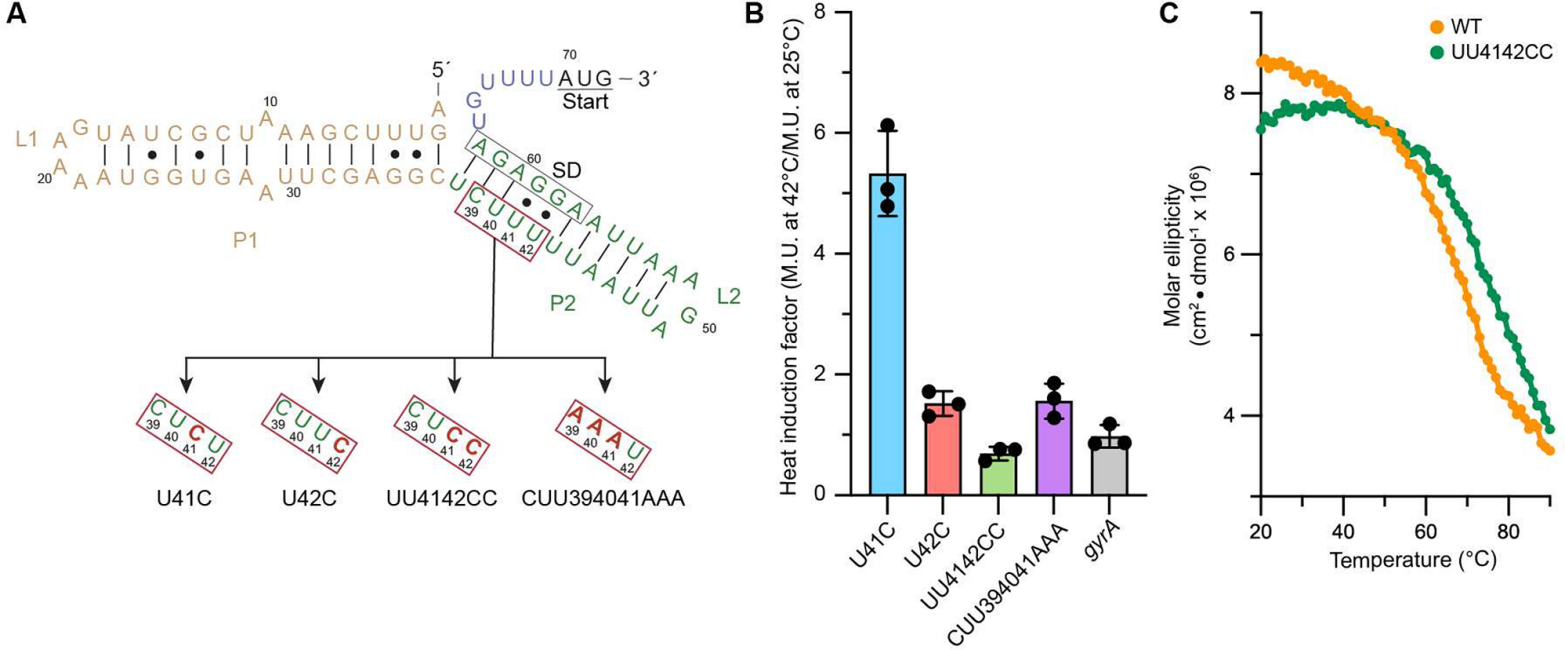
Characterization of the *blyA* 5′-UTR. (A) Secondary structure prediction of the 5′-UTR of *blyA*, showing mutated nucleotides. The start codon is underlined, and the Shine-Dalgarno (SD) sequence is boxed. (B) Heat induction factor of *bgaB* fusions with mutations of the *blyA* 5′-UTR. Expression at 25 and 42 °C of mutants was compared to that of negative control DNA gyrase (*gyrA*) (mean ± standard deviation; *n* = 3 biological replicates). (C) Circular dichroism thermal analysis of the wild-type 5′-UTR of *blyA* (orange) and mutant variant UU4142CC (green) recorded at 290 nm.

The prevailing mechanism through which most RNA thermometers act requires the destabilization of the RNA secondary structure at high temperatures.^19^ At increased temperatures, the stem containing the RBS undergoes a zipper-like, unwinding melting mechanism, allowing access to the RBS.^2,20^ To further investigate the conformational changes induced by heat, structural probing, using 2-methylnicotinic acid imidazolide (NAI) in DMSO, was performed on the *blyA* RNA thermometer sequence. Selective 2′-hydroxyl acylation analyzed by primer extension (SHAPE) provides secondary structure information at a single-nucleotide resolution. The reactivity of each RNA base is correlated with the flexibility of the 2′-OH, with single-stranded or flexible regions exhibiting increased reactivity in opposition to regions engaged in base pairing or other interactions.^21^*In vitro* SHAPE analysis at 25 and 42 °C reveals a prominent temperature-dependent increase in the reactivity of the fourU motif, indicating that the fourU motif is more flexible and accessible in response to an increase in temperature (Figure 3). The most pronounced increases in reactivity were observed in nucleotides U41 and U42, which were shown to be necessary for heat induction (Figure 2B). Interestingly, despite U41 appearing to have the most increased activity at 42 °C, the U41C mutation does not appear to affect translation (Figure 2B). The U41C single mutation is likely not sufficient to stabilize the fourU motif and influence the overall melting temperature of the structure. Overall, structural probing analysis supports a temperature-dependent change in the flexibility and accessibility of the fourU motif of P2, suggesting an increased level of access for ribosome binding at higher temperatures.

**Figure 3:**
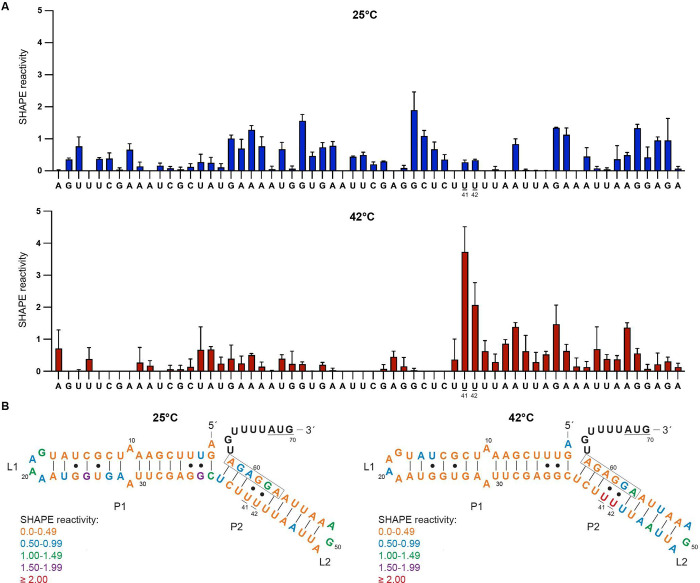
Structural probing analysis of the 5′-UTR of *blyA*. (A) SHAPE reactivity plots for the 5′-UTR of *blyA* at 25 °C (blue) and 42 °C (red) (mean ± standard deviation; *n* = 3 technical replicates). U41 and U42 are underlined. (B) Secondary structure predictions of the 5′-UTR of *blyA* with SHAPE reactivity values at 25 and 42 °C. Nucleotides are color-coded on the basis of the intensity of SHAPE reactivity. U41 and U42 are underlined.

Lastly, to verify if the P2 stem can achieve thermoregulation by itself, a truncated 5UTR_blyA–*bgaB* fusion containing only the P2 stem (mini-5UTR_blyA) (Figure 4A) was tested. Heat induction of mini-5UTR_blyA*–bgaB* fusions resulted in a heat induction factors of ∼3.0- and ∼3.2-fold at 37 and 42 °C, respectively (Figure 4B). Heat induction is notably reduced when P1 is deleted, although P2 is capable of functioning as a thermometer to a lesser extent. In comparison, a similar fusion containing only the P2 hairpin of the *agsA* fourU thermometer retains overall heat induction.^3^ Complete thermoregulation by the *blyA* thermometer is dependent on the P1 and P2 hairpins to accomplish maximal heat induction.

**Figure 4:**
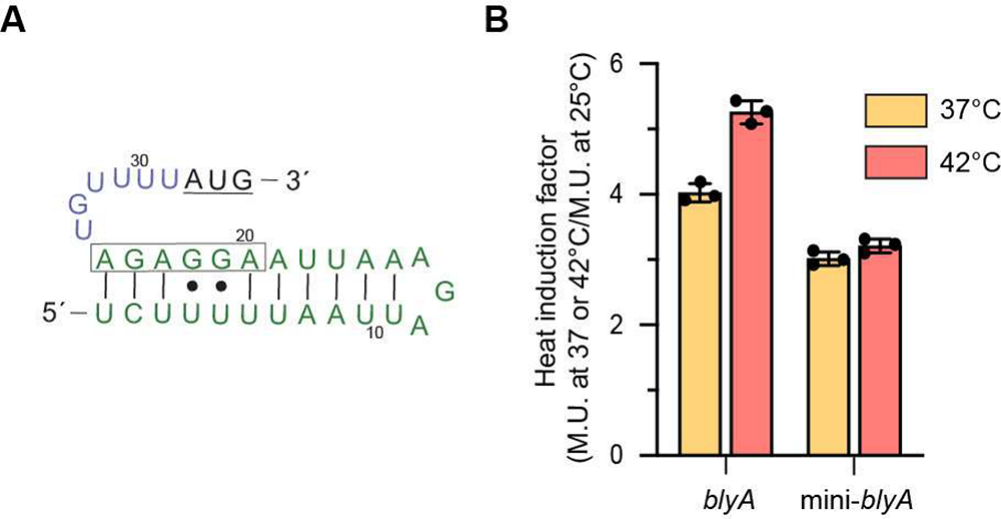
Fold induction of the 5′-UTR of mini-*blyA*. (A) Secondary structure prediction of the truncated 5′-UTR of *blyA* (mini-*blyA*), consisting of only the P2 hairpin. The start codon is underlined, and the Shine-Dalgarno (SD) sequence is boxed. (B) Heat induction factor comparison of *bgaB* fusions with the *blyA* and mini-*blyA* 5′-UTR. Expression was measured at 25, 37, and 42 °C (mean ± standard deviation; *n* = 3 biological replicates).

Although the fourU motif is a common structure found in many RNA thermometers, all previously annotated fourU thermometers have been discovered in bacteria. Additionally, to date, only one other RNA thermometer has been found in a bacteriophage. However, this previously discovered bacteriophage RNA thermometer inhibits gene expression at increased temperatures.^22^ The field of synthetic biology has also shown more interest in RNA thermometers for their potential use in biotechnology and medicine. In this study, we discovered a fourU RNA thermometer located in the 5′-UTR of the *blyA* gene within the genomes of the SPβ prophage and its host, *B. subtilis* 168. The 5′-UTR of the *blyA* gene was identified as an interesting candidate because of its pivotal temperature-dependent role in the SPβ lytic life cycle and because it is widely integrated into the extensively studied *B. subtilis* 168 and has been found in other *B. subtilis* strains.^10−12^ Furthermore, it has been reported that increasing the temperature of the host to 50 °C for a brief period leads to the activation of SPβ through heat induction, causing the phage to replicate and the host cell to undergo lysis. It is postulated that heat induction results in the deactivation of a temperature-sensitive repressor.^10−13^ Our results revealed that the 5′-UTR of the *blyA* gene is an RNA thermometer, indicating an additional level of thermoregulation in SPβ. Our study suggests that similar RNA thermometers may regulate bacteriophage lytic life cycles and may be more common than previously anticipated.

## Supporting Information Available

Experimental procedures, scheme of *bgaB* gene fusion (Figure S1), and temperature-dependent expression of *bgaB* fusions in Miller units (Figures S2–S4) (PDF)

## Abbreviations

UTR: untranslated region
SD: Shine-Dalgarno
SPβ: *B. subtilis* 168 prophage SPβc2
*bgaB*: heat-stable β-galactosidase
*gyrA*: gyrase gene
qRT-PCR: quantitative real-time polymerase chain reaction
SHAPE: selective 2′-hydroxyl acylation analyzed by primer extension
5UTR_blyA: 5′-UTR of *blyA*
mini-5UTR_blyA: truncated 5′-UTR of *blyA*
M.U.: Miller units
