# Supplementary material for: Characterization of a FourU RNA Thermometer in the 5′ Untranslated Region of Autolysin Gene blyA in the Bacillus subtilis 168 Prophage SPβ
Source: Biochemistry. 2023 Sep 12;62(20):2902–7. doi: 10.1021/acs.biochem.3c00368 (PMC10586365; doi:10.1021/acs.biochem.3c00368)
Supplement: Supplementary file 1 — bi3c00368_si_001.pdf [file bi3c00368_si_001.pdf]

Supporting Information

Characterization of a fourU RNA thermometer in the 5'-UTR of autolysin gene *blyA* in the *Bacillus subtilis* 168 prophage SPβ

Alina Y. Tong[1], *Emma E. Caudill*[1‡], *Alexis R. Jones*[1‡], *Luiz F. M. Passalacqua*[2*], *& Michael M. Abdelsayed*[1*]

[1]Department of Biology, California Lutheran University, Thousand Oaks, CA 91360, USA

[2]Laboratory of Nucleic Acids, National Heart, Lung, and Blood Institute, National Institutes of Health, Bethesda, MD 20892, USA

[‡]These authors contributed equally.

*Corresponding authors: luiz.passalacqua@nih.gov and mabdelsayed@callutheran.edu

Experimental procedures

RNA motif search in genomic sequences

Bacterial genomic sequences were downloaded from the NIH National Library of Medicine – National Center for Biotechnology Information using the Nucleotide search (https://www.ncbi.nlm.nih.gov/nuccore/). RNArobo[1] was used to perform RNA motif search utilizing the following descriptor:

s1 h1 s2 h2 s3 h2' s4 h1' s5

s1 0 NNN******

h1 0:0 *NNNNN:NNNNN*

s2 0 N*

h2 0:0 *CUUUUNNNN*:*NNNNAGGAG*

s3 0 NNN****

s4 0 N*

s5 0 NAUG***

Results were manually curated and verified with Basic Local Alignment Search Tool (BLAST) – NIH National Library of Medicine – National Center for Biotechnology Information (https://blast.ncbi.nlm.nih.gov/). Accession number for *Bacillus subtilis 168* is GenBank: CP053102.1. Accession number for *Bacillus subtilis* prophage SPβc2 is GenBank: NC_001884.1.

Plasmid construction

Plasmids were synthesized from VectorBuilder (VectorBuilder Inc, Chicago, IL, USA). The 5'-UTR of *blyA* containing the sequence 5'-AGTTTCGAAATCGCTATGAAAATGGTGAATTCGAGGCTCTTTTTAATTAGAAATTAAGGAGATGTTTTTATG was placed directly upstream of a heat-stable β-galactosidase from *Bacillus stearothermophilus* and driven by a pBAD promoter (pBAD:β-galactosidase). ATG (start codon) in the thermometer sequences replaces the first ATG of *bgaB*. Full vector sequence can be retrieved from the VectorBuilder database using each unique vector ID (https://en.vectorbuilder.com/design/retrieve.html).

Table S1. VectorBuilder IDs

VB ID	Sequence
VB220225-1020jdm	blyA
VB230329-1598bqc	mblyA
VB220714-1515pkn	U41C
VB220714-1516cue	U42C
VB220714-1517afr	UU4142CC
VB220714-1518tey	CUU394041AAA
VB220225-1021dnq	agsA
VB220225-1023wxw	gyrA

RNA synthesis

RNA was synthesized and purchased from the custom RNA Oligo service with RNase Free HPLC purification by Azenta/GENEWIZ (Azenta US, Inc., South Plainfield, NJ, USA). For Circular Dichroism RNA constructs used in this study, RNAs were transcribed from PCR templates (purchased from IDT) with T7 RNA polymerase, and purified by denaturing gel electrophoresis

(10% polyacrylamide, 29:1 acrylamide:bisacrylamide; 1 × TBE, 8 M urea). A 5'-hammerhead ribozyme served to generate 5′-OH termini.[2] After ultraviolet shadowing and excision from gels, RNAs were eluted from the gel into 300 μL of 300 mM KCl and precipitated by adding 700 μL of 100% ethanol at −20 °C.

β-Galactosidase Assays

E. coli DH5α cells carrying *bgaB* plasmids were grown overnight at 25°C in LB broth plus 100 μg/mL ampicillin. Overnight cultures were diluted in LB broth plus 100 μg/mL ampicillin to an optical density at 600 nm (OD600) of 0.05, and then grown at 25°C to an OD600 of 0.3-0.5. Transcription was induced with 0.01% (w/v) arabinose addition, then they were split and incubated at 25, 37, or 42°C. After 30 minutes, 400 μL samples were taken, OD600 was measured, and samples were used for β-galactosidase assays as previously described[3,4] with the following modifications. Three 20 μL samples of culture were added to 80 μL of permeabilization solution (0.8 mg/mL hexadecyltrimethylammonium bromide, 0.4 mg/mL sodium deoxycholate, 100 mM Na_2HPO_4, 20 mM KCl, 2 mM $MgSO_4$, and 5.4 μL/mL β-mercaptoethanol). After a 30-minute incubation at 30°C, 600 μL of substrate solution (60 mM Na_2HPO_4, 40 mM NaH_2PO_4, 1 mg/mL o-nitrophenyl-β-D-Galactoside (ONPG), 2.7 μL/mL β-mercaptoethanol) was added. The reactions were incubated at 55°C for 1-2 hours. The addition of 700 uL of 1 M Na_2CO_3 terminated the reactions to be prepared for absorbance readings. Assays were performed in triplicate. Heat induction factor is calculated by dividing expression in Miller Units at 37 or 42°C by expression at 25°C.

Quantitative real time-PCR (qRT-PCR)

Samples for comparative qRT-PCR and β-galactosidase assays were taken from the same cultures post-incubation at 25 or 42°C. RNA was isolated, treated with DNAse I, and purified using Direct-zol RNA Miniprep kit (Zymo). After purification, 300 ng RNA were used for reverse transcription reactions performed using SuperScript IV Reverse Transcriptase kit (Invitrogen) according to manufacturer's instructions. cDNA was amplified and detected in the Applied Biosystems StepOne Real-Time PCR System, using the PowerTrack SYBR Green Master Mix (ThermoFisher) and specific primers (Table S2) for *bgaB* and *gyrA*. Assays were performed with three biological replicates, each with three technical triplicates. Relative *bgaB* transcript amounts were calculated using the ΔΔCt method,[5] and experimental Ct values were normalized to the non-thermoregulated reference gene *gyrA*.

Circular Dichroism

Circular dichroism (C.D.) data were recorded in an Applied Photophysics Chirascan Q100 Spectrometer using a 0.1 mm flow cell. Folded samples containing 25 μM RNA in buffer (10 mM Tris-HCl pH 7.5, 50 mM KCl, 2 mM $MgCl_2$) were analyzed for thermal analysis with smooth ramp mode at ramp-rate of 1ºC per minute from 20ºC to 90ºC. Five different wavelengths (286, 288, 290, 292, and 294 nm) were used in the thermal analysis. Data were analyzed using Global 3 Thermal Analysis Software (Applied Photophysics).

Selective 2′-Hydroxyl Acylation and Primer Extension (SHAPE)

SHAPE experiments were performed as described previously with the following modifications.[6,7] SHAPE reactions were prepared in a total volume of 10 μL. 10 pmol of purified RNA were denatured to 95°C in water for 5 min, then snap cooled on ice. Then RNA was added to a buffer containing 140 mM KCl, 10 mM NaCl, 10 mM tris chloride, 1 mM $MgCl_2$ at pH 7.4, and incubated at room temperature for 10 minutes. Then, 30 mM 2-Methylnicotinic acid imidazolide in DMSO (NAI, Sigma-Aldrich, CAS number: 1055970-47-2) was added to the mixture (+ reaction) or 10% DMSO (- reaction), and the reaction was incubated for 25 min at either 25 or 42°C. Reactions were precipitated with 10 μL 3 M KCl, 1 μL glycoblue, 89 μL H_2O, and 300 μL 98% ethanol. Reactions were performed in technical triplicates.

The RNA pellet was reconstituted in a 20 μL reaction volume containing 0.1 μM of 5' labeled 6-FAM reverse transcription DNA primer, 4 μL of 5 × SSIV Buffer, 200 U SSIV Reverse Transcriptase (Thermo Fisher), and 1 mM each of deoxyribonucleotide triphosphate. Extensions were performed at 50°C for 15 min, then activated by heating at 80°C for 10 minutes. 5 μL of 400 mM NaOH was added, and the reaction was incubated at 95°C for 5 min to hydrolyze the RNA. Reactions were precipitated with 10 μL of 3 M KCl, 1 μL of glycoblue, 89 μL of H_2O, and 300 μL of 98% ethanol.

Complementary DNA (cDNA) was reconstituted in 10 μL H_2O. Capillary electrophoresis was performed by Azenta/GENEWIZ (Azenta US, Inc., South Plainfield, NJ, USA) using the Fragment Analysis service. cDNA was resolved with Applied Biosystems 3730xl Genetic Analyzers. ABI DS33 Dye Set/Liz 500 Ladder was supplied by Azenta/GENEWIZ. SHAPE experiments from

three independent experiments were analyzed by the RiboCAT (Ribonucleic acid Capillary-electrophoresis Analysis Tool)[8] SHAPE analysis software.

Table S2. Sequences of the synthesized oligonucleotides

Oligonucleotide	Description	Sequence
blyA[a]	Sequence for β-galactosidase assay	5'-AGTTTCGAAATCGCTATGAAAATGGTGAATTCGAGGCTCTTTTTAATTAGAAATTAAGGAGATGTTTTTATG
mini-*blyA*[a]	Sequence for β-galactosidase assay	5'-TCTTTTTAATTAGAAATTAAGGAGATGTTTTTATG
U41C[a]	Sequence for β-galactosidase assay	5'-AGTTTCGAAATCGCTATGAAAATGGTGAATTCGAGGCTCT**C**TTTAATTAGAAATTAAGGAGATGTTTTTATG
U42C[a]	Sequence for β-galactosidase assay	5'-AGTTTCGAAATCGCTATGAAAATGGTGAATTCGAGGCTCTT**C**TTAATTAGAAATTAAGGAGATGTTTTTATG
UU4142CC[a]	Sequence for β-galactosidase assay	5'-AGTTTCGAAATCGCTATGAAAATGGTGAATTCGAGGCTCT**CC**TTAATTAGAAATTAAGGAGATGTTTTTATG
CUU394041AAA[a]	Sequence for β-galactosidase assay	5'-AGTTTCGAAATCGCTATGAAAATGGTGAATTCGAGGCT**AAA**TTTAATTAGAAATTAAGGAGATGTTTTTATG
agsA[a]	Sequence for β-galactosidase assay	5' TGTAGTCAGTGGACAAGCAATGCTTGCCTTGATGTTGAACTTTTGAATAGTGATTCAGGAGGTTAATGATG
gyrA[a]	Sequence for β-galactosidase assay	5'-ACCTCAAACTGCGCGGCTGTGTTATAATTTGCGACCTTTGAATCCGGGATACAGTAGAGGGATAGCGGTTAGATG
SHAPE RNA *blyA*	RNA for SHAPE	5'-AGUUUCGAAAUCGCUAUGAAAAUGGUGAAUUCGAGGCUCUUUUUAAUUAGAAAUUAAGGAGAUGUUUUUAUGUCAGUUUUCACUAAUAGCUACAUU
SHAPE rev primer	SHAPE reverse primer	5'-6-FAM-AATGTAGCTATTAGTG
gyrA fwd primer	qPCR primer	5'-GTCAGGGTAACTTCGGTTCTATC
gyrA rev primer	qPCR primer	5'-GAGATCGGCCATCAGTTCAT
bgaB fwd primer	qPCR primer	5'-CCGTGGTGCAGATGGTATTAT
bgaB rev primer	qPCR primer	5'-CTCTTGCCCTAACTGTGTAACT
C.D. WT *blyA*[a]	Sequence for C.D.	5'-GTTTCGAAATCGCTATGAAAATGGTGAATTCGAGGCTCTTTTTAATTAGAAATTAAGGAGATGTTTTTATG
C.D. UU4142CC[a]	Sequence for C.D.	5'-GTTTCGAAATCGCTATGAAAATGGTGAATTCGAGGCTCT**CC**TTAATTAGAAATTAAGGAGATGTTTTTATG

[a] underlined ATG is the start codon for *bgaB*

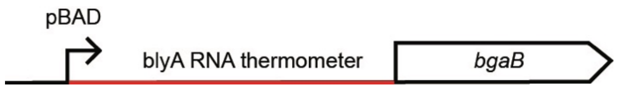

Figure S1. Scheme depicting *bgaB* gene fusion with pBAD promoter system as used for β-galactosidase assays.

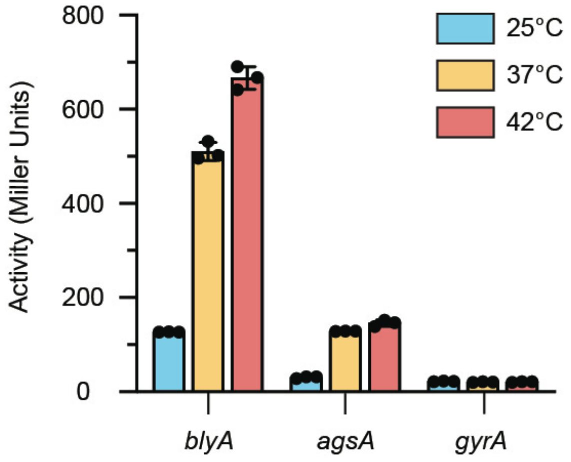

Figure S2. Temperature-dependent expression of *bgaB* fusions with the *blyA* 5'-UTR. Expression in Miller Units at 25, 37, and 42°C was compared to a positive control of the *agsA* fourU RNA thermometer as well as a negative control DNA gyrase (*gyrA*). (mean ± s.d., n = 3 biological replicates) (See Figure 1D for data shown as heat induction factor).

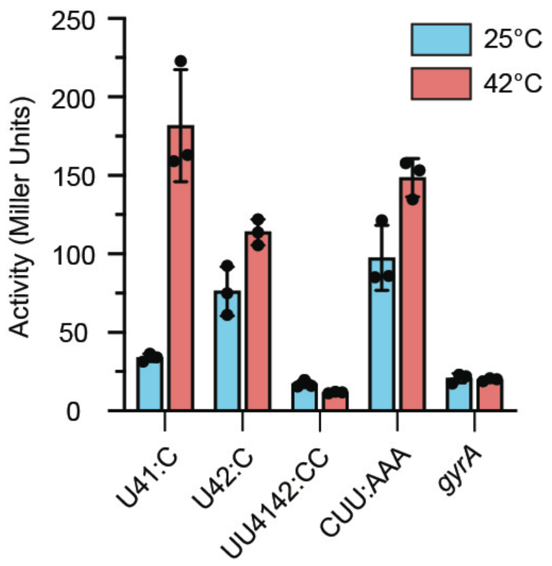

Figure S3. Temperature-dependent expression of *bgaB* fusions with mutated variants of the *blyA* 5'-UTR. Expression in Miller Units at 25 and 42°C of mutants were compared to negative control DNA gyrase (*gyrA*) (mean ± s.d., n = 3 biological replicates) (See Figure 2A for data shown as heat induction factor).

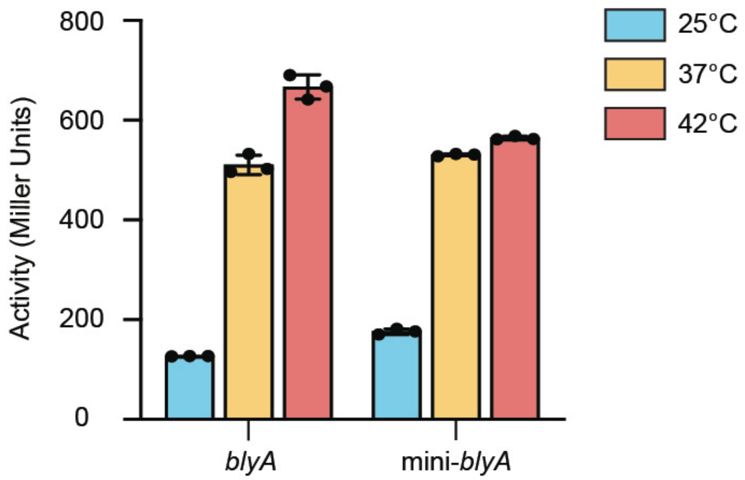

Figure S4. Temperature-dependent expression of *bgaB* fusions with the mini-*blyA* 5'-UTR. Expression in Miller Units at 25, 37, and 42°C was compared to the full-length *blyA* 5'-UTR (mean ± s.d., n = 3 biological replicates) (See Figure 4B for data shown as heat induction factor).

References

(1) Rampášek, L.; Jimenez, R. M.; Lupták, A.; Vinař, T.; Brejová, B. RNA motif search with data-driven element ordering. *BMC Bioinform.* **2016**, *17* (1), 216. DOI: 10.1186/s12859-016-1074-x.

(2) Price, S. R.; Ito, N.; Oubridge, C.; Avis, J. M.; Nagai, K. Crystallization of RNA-protein complexes I. Methods for the large-scale preparation of RNA suitable for crystallographic studies. *J. Mol. Biol.* **1995**, *249* (2), 398–408. https://doi.org/10.1006/jmbi.1995.0305.

(3) Miller, J. H. *Experiments in Molecular Genetics*; Cold Spring Harbor Laboratory Press: Cold Spring Harbor, NY, 1972.

(4) Zhang, X.; Bremer, H. Control of the *Escherichia coli* rrnB P1 promoter strength by ppGpp*. *J. Biol. Chem.* **1995**, *270* (19), 11181–11189. DOI: 10.1074/jbc.270.19.11181.

(5) Livak, K. J.; Schmittgen, T. D. Analysis of relative gene expression data using real-time quantitative PCR and the 2−ΔΔCT method. *Methods* **2001**, *25* (4), 402–408. DOI: 10.1006/meth.2001.1262.

(6) Wilkinson, K. A.; Merino, E. J.; Weeks, K. M. Selective 2′-hydroxyl acylation analyzed by primer extension (SHAPE): Quantitative RNA structure analysis at single nucleotide resolution. *Nat. Protoc.* **2006**, *1* (3), 1610–1616. DOI: 10.1038/nprot.2006.249.

(7) Choi, E. K.; Ulanowicz, K. A.; Nguyen, Y. A. H.; Frandsen, J. K.; Mitton-Fry, R. M. SHAPE analysis of the *htrA* RNA thermometer from *Salmonella enterica*. *RNA* **2017**, *23* (10), 1569–1581. DOI: 10.1261/rna.062299.117.

(8) Cantara, W. A.; Hatterschide, J.; Wu, W.; Musier-Forsyth, K. RiboCAT: A new capillary electrophoresis data analysis tool for nucleic acid probing. *RNA* **2017**, *23* (2), 240–249. DOI: 10.1261/rna.058404.116.
